# Collaborative Surveillance: Using a Minimum Set of Key Data Parameters for One Health Participatory Surveillance

**DOI:** 10.2196/77448

**Published:** 2025-08-08

**Authors:** Mark Smolinski, Nomita Divi, Onicio Leal Neto

**Affiliations:** 1 Ending Pandemics Academy Mel and Enid Zuckerman College of Public Health University of Arizona Tucson United States

**Keywords:** One Health, participatory surveillance, collaborative surveillance, data parameters, pandemics, epidemics, global health security

## Abstract

Early detection of a newly emerging or reemerging infectious disease is crucial to minimize the impact of such a threat on lives and livelihoods. With three of four pathogens capable of causing epidemics or pandemics arising first in animals and spreading to humans as zoonosis, a One Health approach to early detection is paramount. One Health participatory surveillance, defined as the bidirectional receiving and transmitting of data for action through direct engagement of the target population, is an effective form of collaborative surveillance to enhance global health security. Participatory surveillance systems can vary greatly when developed for a specific purpose or to meet a particular community’s needs. Different geographies, languages, customs, beliefs, and practices often influence the breadth and depth of the data collected within each system. Imagine, however, if each of these varied systems could “speak” to each other, sharing their aggregated, deidentified data to create a comprehensive, real-time view of planetary health. The key is to collect the same information from users in each system, or at least a minimum set of key data parameters, to generate One Health surveillance greater than that of any individual system. To enable this vision, we propose a minimum set of key data parameters for One Health participatory surveillance that could be collected in any system through self-reporting by the public. This real-time collaborative surveillance could be the earliest indicator of a human, animal, or environmental health threat as it does not require interaction with a health care facility or provider where most disease surveillance traditionally occurs. One Health participatory surveillance that can detect major syndromes of potential emerging or reemerging pathogens through self-reporting on human, animal, or environmental health is a practical, scalable solution to identify and respond to rapidly spreading contagions.

## Introduction

Of infectious diseases with epidemic or pandemic potential, 75% are zoonoses, meaning they spread to humans from wildlife or domestic animals [1,2]. Oftentimes, insect vectors such as mosquitoes, ticks, or fleas play a pivotal role in spreading disease from animals to humans [3,4]. Other times, infections spread from an infected animal to humans by way of direct contact with skin or saliva, or via the respiratory route [5]. In the case of hantavirus, for example, the inhalation of virus-contaminated particles when sweeping floors covered with dried urine and feces from deer mice led to acute respiratory infections in humans [6-8]. Regardless of the method of spillover, once a pathogen is established within the human population, numerous factors such as attitudes, behaviors, demographics, and politics can often exacerbate the further spread among humans [9,10].

**Figure 1:**
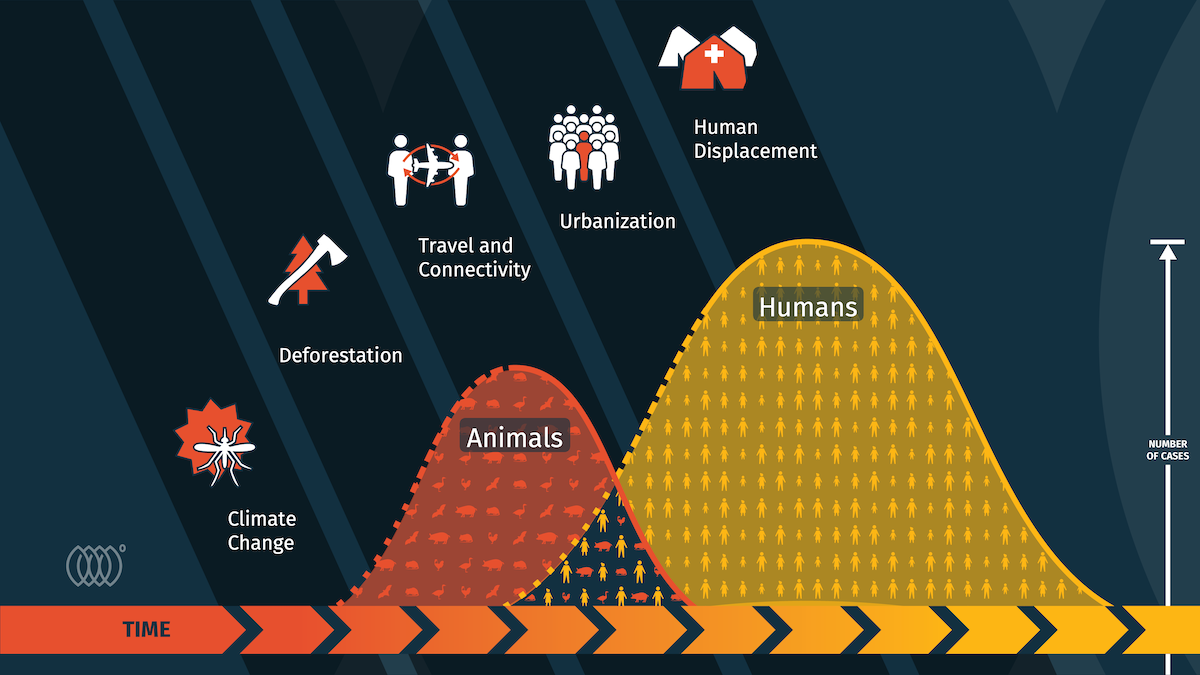
A One Health approach. Of every newly emerging infectious disease, 75% arise in animals and spread to humans. Animal outbreaks (shown in red) can quickly lead to human outbreaks (shown in yellow) when people are in close contact with livestock or wildlife. If we prioritize our efforts to find outbreaks faster in animals, it is possible to prevent human infection. Factors like climate change, urbanization, and increased travel and trade can exacerbate the spread and must also be considered.

Various additional dynamics lead to the emergence and spread of infectious diseases, including the speed and extent of international travel and trade, land use, climate change, lack of public health infrastructure, and access to health care, to name just a few (Figure 1) [9,11]. Although not all drivers of disease emergence can be eliminated, cooperation and collaboration across public and private sectors, scientific disciplines, and geographic borders can significantly improve early detection and trigger an effective and timely response. An integrated approach to disease surveillance across species is paramount for the early detection and prompt response to emerging infections [12]. Prioritizing surveillance in planetary hot spots, those areas with the greatest likelihood of zoonotic outbreaks, is a compelling approach to reducing collective risk [13].

## One Health Participatory Surveillance

Directly engaging communities to report on the health of their family, themselves, and their animals, and any concerns regarding air or water quality, or other environmental health hazards is how we can safeguard the world from epidemics and pandemics. Nobody understands that something is “not quite right” better than the person who lives, works, or frequently spends time where these observations are occurring, and of course, nobody understands something is not right in how a person feels better than the individual themself.

Imagine if each of us took a couple of minutes each day, or even just once per week, to report on our health status and our family’s, or concerns within our immediate environmental surroundings. This could be done through email prompts, smartphone apps, brief SMS text messages, social media posts, or an interactive voice response system, or even seamlessly via passive data already captured by our phones, wearable devices, or other smart technologies. Ideally, if the majority of the community were doing the same, this form of collaborative surveillance [12] could detect the earliest signs of an emerging health threat. Everyone’s report would anonymously be connected on a visual platform to “see” that something is not right in a particular geographical region or simultaneously in several regions at a given point in time. This real-time collaborative surveillance could be the earliest indicator of a human, animal, or environmental health threat, as it does not require an interaction with a health care facility or provider, where most disease surveillance traditionally occurs.

Speed of detection is crucial. The faster a threat is identified and verified as true, the faster prompt actions can ensue to minimize its impact. Faster action saves lives and preserves livelihoods [14,15]. Once a threat gets a foothold in society, its impact grows exponentially in most cases. This is especially true for infectious disease threats once they enter the human population.

Collaborative surveillance can be achieved through participatory surveillance, defined as the process of receiving and transmitting data for action through direct engagement of the target population [16,17]. Participatory surveillance is the most efficient, cost-effective, and inclusive method to detect threats in real time—anywhere and everywhere across the world [18-21]. Each participatory surveillance system can be designed in any language, engaging users through local customs, practices, and beliefs, and adding features such as gamification to attract certain age groups or specific cohorts. Recruiting and retaining users is a significant obstacle that can be overcome with user-centric design principles and other nonfinancial incentives [22,23]. The more users that are engaged with the system, the more useful it becomes for early detection. The 2-way communication enabled through this approach can also be instrumental in mounting a rapid and effective response. Numerous COVID-19–specific self-reporting systems were developed during the pandemic, validating the utility of participatory surveillance for the early detection and monitoring of emerging infections [24-26]. As more ecosystem dynamics and environmental data are integrated with human and animal disease surveillance efforts, the likelihood of accurately predicting and preventing zoonotic disease outbreaks will increase.

The most effective disease surveillance requires communities to harmoniously apply the best science, technology, and practices regarding human, animal, and environmental health in planetary hot spots for emerging infections and beyond. For the past decade, Ending Pandemics, a US-based nonprofit, has provided technical guidance and catalytic funding to support participatory surveillance in partnership with low- and middle-income countries on five continents [27]. A stellar example of One Health participatory surveillance was created in 2014 in Chiang Mai, Thailand, where community health volunteers are trained to report suspected illness in domestic livestock and poultry through submission of photos and simple data forms on mobile phones. Today, the system is receiving reports on food safety, contaminated water, and counterfeit drug sales in addition to reports of human or animal illness. In 2025, the system added wastewater surveillance as another mechanism for the early detection of an emerging zoonosis [28]. This One Health participatory approach was replicated in Tanzania in 2016, where village health volunteers report on human and animal illnesses among the pastoral herding communities whose livestock often comingle with the wildlife in the Serengeti region. Participatory surveillance has also been successfully used during mass gatherings in Brazil with the 2014 World Cup and 2016 Olympic Games [19]. The most successful participatory surveillance systems are those that are collaboratively developed, locally owned and resourced, and established as deep partnerships with the government from the time of inception.

## Expanding Collaborative Surveillance

Although individual participatory surveillance systems exist, imagine if each of these varied systems could “speak” to each other, sharing their aggregated, deidentified data to expand the global view of the planet’s health. Each of these systems can be designed to engage specific population groups, churches, schools, workplaces, or any other social network. The key is to collect the same information from users in each system, or at least a minimum set of key data parameters, to generate One Health surveillance greater than that of any individual system.

Perhaps an analogy would be useful. If each surveillance system represents a spoke on a wheel, then each system has a pivotal role in holding the wheel’s rim in place and distributing pressure evenly. For the wheel to turn smoothly and bear weight without collapsing, every spoke must be made of the right material, cut to the correct length, and fastened securely. If even one spoke is too weak, too short, or poorly connected, the integrity of the entire wheel is compromised. The more spokes you have, the stronger and more resilient the wheel becomes. This redundancy adds durability and performance, allowing the wheel to handle greater challenges without faltering. If the wheel itself represents global disease surveillance, then each spoke signifies a vital part of this global system by capturing key information at the local, national, or regional levels.

**Figure 2:**
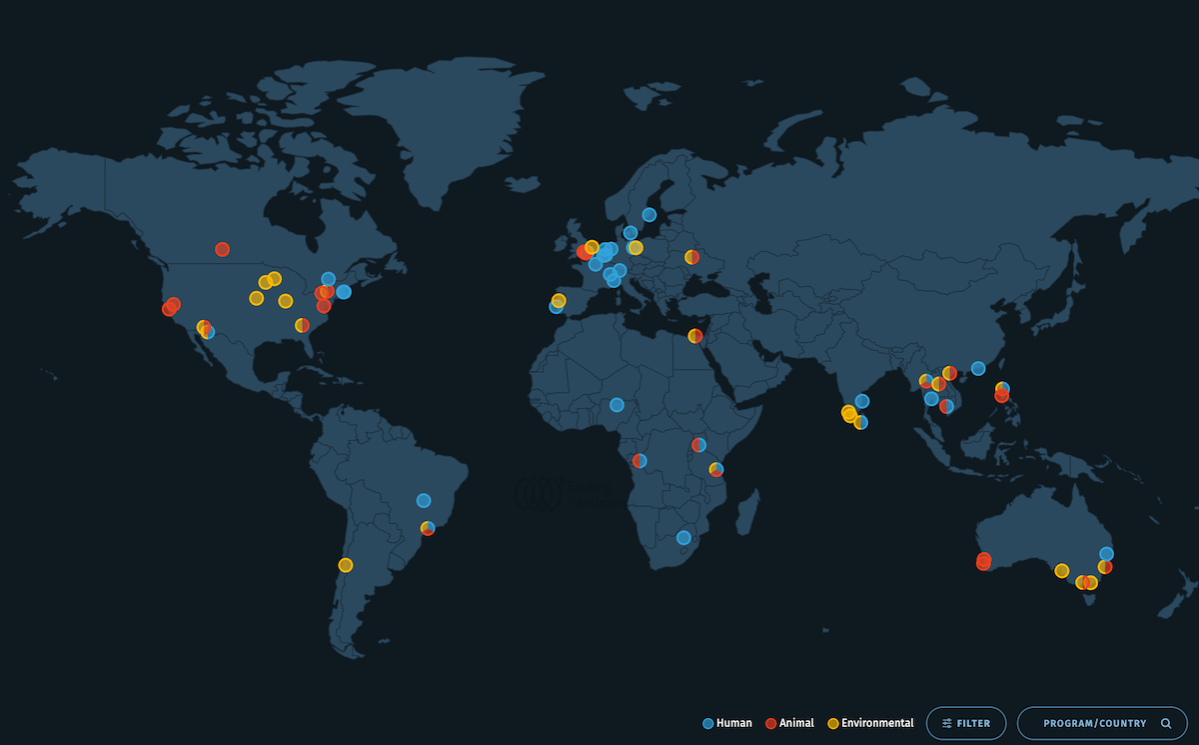
One Health participatory surveillance interactive landscape map. This interactive map highlights participatory surveillance systems in people, animals, and ecosystems for detecting and responding to potential disease outbreaks. The systems’ colors indicate the sector they focus on, with red indicating animal health, blue indicating human health, and yellow indicating environmental health.

To get a better idea of progress toward creating this global disease surveillance “wheel,” in 2022, Ending Pandemics conducted a systematic literature review and surveys to map the participatory surveillance “spokes” monitoring the health of animals, humans, or the environment across the world (Figure 2) [17,29]. The resulting map illustrates that the approach of directly engaging communities in self-reporting the health status of their members, animals, or environment is rapidly increasing in all areas of the world. Users of this map can learn key details about each system, including the number of reporters, the focus area of the system, and key contacts for the creators of the system. Although every system on the map represents bidirectional information sharing, some are sector specific and limit their reach to wildlife, livestock, the environment, or human health monitoring. Several systems, however, have achieved the One Health monitoring goal of incorporating data parameters that span all sectors. One Health participatory surveillance is the key to quickly finding and responding to emerging health threats and, in some cases, is contributing to the prediction and prevention of potential diseases with epidemic or pandemic potential. The ultimate success of One Health participatory surveillance will be realized when all systems can share data as mutual key data parameters are collected among the various systems.

## One Health Participatory Surveillance Data Parameters Compendium

Every participatory surveillance system collects information as a set of data parameters. Ending Pandemics compiled a compendium of data parameters from participatory surveillance systems that operate in one or more of the One Health sectors: humans, animals, and the environment. Through a series of virtual workshops in 2021-2022 with experts from each sector, including health experts from the World Health Organization, the Food and Agriculture Organization of the United Nations, and the World Organization for Animal Health, a set of data parameters within each sector was compiled that were geared toward the public. Data parameters that seemed too “specialized” for public participation were excluded from the final set. Where available, information was gathered on how different parameters are collected within the individual systems.

The One Health Participatory Surveillance Data Parameters Compendium [30] is a snapshot into the world of participatory surveillance systems collecting data on human, animal, and environmental health. The compendium illustrates the breadth and depth of what is being used in participatory surveillance systems operating across the world. The compendium can serve the needs of those who may be contemplating the development of a participatory surveillance system, looking to expand a current system to include additional health sectors, or adapting data parameters to match other systems to foster collaborations and data sharing.

## Establishing a Minimum Set of Key Data Parameters

Participatory surveillance systems are generally designed to collect information relevant to the community it is monitoring with a specific intent or purpose. Hence, some systems are focused directly on the public’s health, and others might be tailored more to wildlife health or conservation, with the “reporter” having special training or expanded knowledge relative to the system’s intent, such as a park ranger or veterinarian. Imagine, however, if we could collect a minimum set of key data parameters that monitor One Health across every participatory surveillance system, not necessarily all the parameters in each system, but a minimum set of key data parameters that can detect an emerging health threat of zoonotic origin by monitoring the health of animals, humans, and the environment.

**Figure 3:**
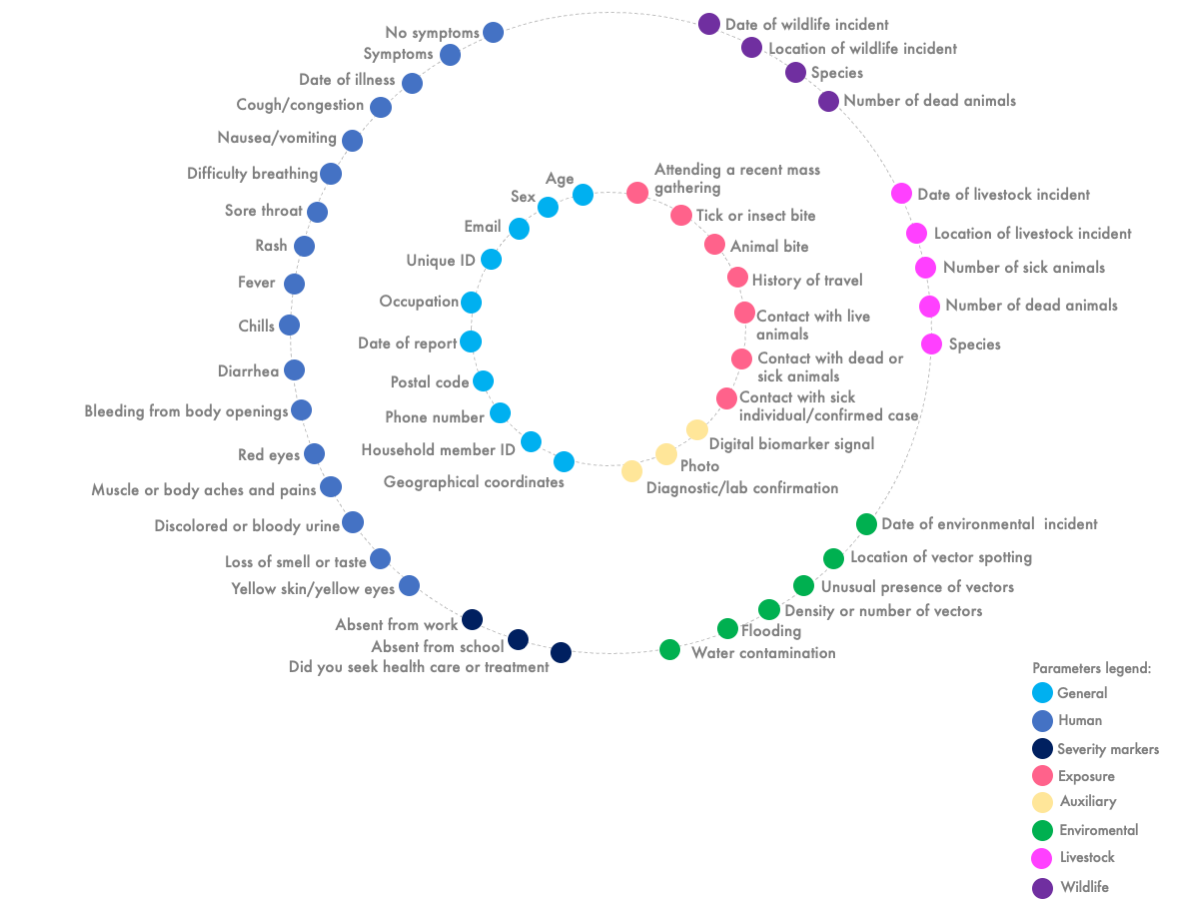
Minimal set of key data parameters for One Health participatory surveillance. This set of 42 parameters across human, animal, and environmental health, along with 10 general parameters and additional auxiliary parameters, can be incorporated into participatory systems to enable One Health surveillance to detect any emerging zoonosis.

Ending Pandemics used the compendium of data parameters collected across the known participatory surveillance systems and reduced this fairly massive set of data parameters to approximately 100 data parameters for consideration in a system designed for use by any member of a community (ie, the public). In 2022, the Fourth International Workshop on Participatory Surveillance (IWOPS) convened human, animal, and environmental health experts from 28 countries to further refine this set of 100 parameters into a potential minimum set of key data parameters for inclusion in any system [31]. The main goal of this intensive consultation process was the ability of this minimum set of key data parameters to detect any emerging zoonosis, with the ultimate goal of potentially detecting environmental, wildlife, and domestic livestock and poultry threats before any human illness. If not detected before spillover, this minimum set of key data parameters can provide a fuller picture of an emerging infectious disease threat as the earliest human cases are identified through self-reporting. Our proposed minimum set of key data parameters consists of 42 data parameters across human, animal, and environmental health, along with 10 general data parameters to collect information on demographics, location, and timing of reports. We have also included the potential for photographs, diagnostic tests or laboratory confirmation, and digital biomarkers (eg, automated wearables, contactless physiological sensing, or other smart technologies) to be added by system designers where relevant and available (Figure 3). The addition of these latter data elements can add specificity to the system and help validate the self-reported syndromic data.

## The Value of a Minimum Set of Key Data Parameters

As stated earlier, the faster an emerging infection is detected, the quicker an effective response can ensue to limit loss of life and preserve livelihoods. Several major epidemics and pandemics throughout history were the result of a previously unrecognized pathogen [32]. Hence, relying on diagnostics or laboratory tests would not be useful at the earliest stages of such outbreaks. In fact, with the lack of readily available, widely dispersed diagnostics for even known pathogens, the clustering of similar symptoms in time and space is often the only means to detect an emerging disease. Syndromic surveillance, therefore, is often relied upon for the early detection of a newly emerging or reemerging infectious disease [33]. Participatory surveillance that can detect major syndromes of potential emerging or reemerging pathogens through self-reporting of illness is a practical way to help identify a rapidly spreading contagion.

In general, the minimum set of key data parameters should identify a newly emerging or reemerging pathogen based on the frequency of reports and the geographical location of such reports. We believe that respiratory diseases have the highest likelihood of wide-scale epidemics or pandemics, and the minimum set of key data parameters should pick up such a threat. Cough and shortness of breath, plus or minus sore throat, can be effective at identifying influenza, severe acute respiratory syndrome, Middle East respiratory syndrome, or a yet unknown respiratory pathogen. When combined with fever, malaise, body aches or pains, a cluster of such reports heightens concerns of a serious respiratory threat. It may even be possible to specify a potential diagnosis with additional symptoms being reported. Likewise, data parameters on health-seeking behavior and absenteeism from school or work can provide further evidence of illness severity.

Additional self-reported symptoms can also identify other potential pathogens by collecting syndromic information on rash illness, diarrheal illness, or hemorrhagic fever. Key data parameters on exposure to domestic animals, wildlife (including rodents), and insect vectors like mosquitoes, ticks, and fleas can lead to a plethora of other zoonotic threats to be considered as the cause of an outbreak. Furthermore, several of the key data parameters in the minimum set can provide valuable information on sickness or die off in domestic animals or wildlife, or environmental concerns like flooding or water safety that might spur further investigation, leading to the prediction and prevention of human illness from zoonosis.

The minimum set of key data parameters was analyzed by expert human curation to validate that most emerging infectious disease threats with epidemic or pandemic potential could be identified through various combinations of terms. For example, the minimum set of key data parameters should be able to identify most emerging infections capable of causing epidemic or pandemic disease. The World Health Organization updated its list of priority pathogens capable of causing epidemic and pandemic diseases in 2024 [34] by convening over 300 scientists to consider the evidence on virus families and bacteria, as well as “Disease X.” Disease X is included to indicate an unknown pathogen that could cause a serious epidemic or pandemic. With major syndromes being reported regularly, it is more than likely that Disease X would also be detected through One Health participatory surveillance.

## The Value of Collecting Similar Data Parameters: Case of Global Flu View

Participatory surveillance systems for influenza-like illness have been well established in numerous countries, many operating for over a decade [35]. Ending Pandemics convened the developers of each of these systems at the Second IWOPS in 2013 [36]. The IWOPS community established a minimum set of data parameters among systems collecting self-reported information to monitor the annual risk of influenza. As a result, deidentified and aggregated data are shared on a digital platform, Global Flu View (GFV), to improve situational awareness and increase our understanding of how influenza emerges across the world [37].

GFV is a system orchestrator platform that converges and combines influenza-related data from 11 countries, harmonizing the way information is captured and reported by ensuring that datasets from different systems and regions remain consistent, comparable, and accurate. As a part of the platform’s approach, GVF offers a ready-to-use tool kit for institutions interested in deploying or enhancing their own participatory surveillance systems. The tool kit significantly lowers the barriers to entry by providing a clear and concise guide on the key data parameters that have already been vetted across multiple regions. At the same time, GFV maintains a high degree of flexibility to accommodate local realities and linguistic nuances, allowing the naming and coding of parameters to be adapted into more culturally and contextually appropriate terms. Ultimately, this approach amplifies the quality and timeliness of influenza-like illness data worldwide, helping health authorities spot potential outbreaks sooner and collaborate more effectively in responding to public health threats.

GFV represents one example of what is possible when participatory surveillance systems can “speak” to each other regarding influenza-like illness data parameters. Embracing the entire minimum set of key data parameters encompassing One Health will expand the value of participatory surveillance for all epidemic and pandemic threats.

## Limitations

This minimum set of key data parameters is not without limitations. The parameters are based on syndromic surveillance, with the goal of being highly sensitive to enable early detection of any potential health threat to the community. A primary goal of participatory surveillance is speed of detection; hence, the system may produce false positives. The occurrence of false positives, however, is offset by the potential to quickly uncover a rapidly spreading infection within the population. Measuring sensitivity generally requires comparison with “gold standard” surveillance data to determine the true frequency of the disease in the population [38]. To capture the sensitivity of a particular participatory surveillance system, a gold standard for early detection will need to match the purpose of this system. While the broad range of parameters in this minimum dataset provides high sensitivity, the parameters lack specificity. This reduced specificity may be overcome as participatory surveillance systems allow users to include diagnostic or laboratory tests, digital biomarkers, or other data derived from future advances in technology like wearables and environmental sensors. Furthermore, as the field of rapid diagnostics continues to expand in all areas of the world, especially the availability of home test kits, greater specificity in diagnosing the threat among the population reporting into the system will be possible. Further work is needed to determine the sensitivity and specificity of the minimum set of key data parameters for One Health participatory surveillance.

The minimum set of key data parameters is intended to rapidly detect any emerging zoonosis, whether signs and symptoms appear first in animals or humans. As such, the minimum set of key data parameters may not detect some endemic diseases or subclinical infections in humans and animals, nor are they intended to monitor the general health of the population. Participatory surveillance is intended to complement other surveillance efforts such as severe acute respiratory infection, sentinel sero-surveillance, sequential biologic sampling in high-risk communities, or health care–based surveillance systems. Integrating participatory surveillance among populations that are also part of the severe acute respiratory infection or other targeted surveillance systems can serve to expand the reach of monitoring within a given population.

## Conclusion

Early detection is paramount when it comes to any emerging infectious disease. Participatory surveillance is an effective method of collaborative surveillance that allows anyone to participate in monitoring the health of their communities to detect the earliest signs of an emerging infectious disease threat. Furthermore, participatory surveillance allows for monitoring factors in the environment along with wildlife and domestic livestock surveillance for detecting zoonotic diseases. A minimum set of key data parameters for One Health participatory surveillance can enable an individual system anywhere in the world to connect to share information with other systems collecting similar data. This, of course, requires standardization of data collection or analysis to permit true comparisons among systems. As more systems become integrated into regional or global surveillance efforts, the greater the chances of detecting zoonotic diseases of epidemic or pandemic potential at their earliest stages of emergence. Persons or organizations using the minimum set of key data parameters to create a new participatory surveillance system or to expand a system currently in use can adapt these parameters to fit the needs of their specific community. For example, they can survey users within their system to help ascertain the applicability and clarity of the specific parameters and the potential need for revisions or additional terms. With further advances in environmental and animal health monitoring, it may be possible to predict and prevent human infections from occurring at all. When this potential is realized, the resulting greatly enhanced global health security will allow the prevention of major epidemics and pandemics.

## Abbreviations

GFV: Global Flu View
IWOPS: International Workshop on Participatory Surveillance
